# Adaptive Bandelet Transform and Transfer Learning for Geometry-Aware Thyroid Cancer Ultrasound Classification

**DOI:** 10.3390/diagnostics16040554

**Published:** 2026-02-13

**Authors:** Yassine Habchi, Hamza Kheddar, Mohamed Chahine Ghanem, Jamal Hwaidi

**Affiliations:** 1 Faculty of Technology, University Salhi Ahmed, Naama 45000, Algeria; 2 LSEA Laboratory, Department of Electrical Engineering, University of Medea, Medea 26000, Algeria; 3 School of Computer Science and Informatics, University of Liverpool, Liverpool L69 3BX, UK; 4 Department of Engineering, City St George, University of London, London EC1V 0HB, UK

**Keywords:** bandelet transform, transfer learning, thyroid cancer, deep learning, medical imaging, diagnostic

## Abstract

**Background and Objectives:** Classification of thyroid nodules (TN) in ultrasound remains challenging due to limited labelled data and the limited capacity of conventional feature representations to capture complex, multi-directional textures. This work aims to improve data-efficient TN classification by integrating a geometry-adaptive Bandelet Transform (BT) with transfer learning (TL) to enhance feature representation and generalisation. **Methods:** The proposed pipeline first applies BT to strengthen directional and structural encoding in ultrasound images via quadtree-driven geometric adaptation. It then mitigates class imbalance using SMOTE and increases data diversity through targeted data augmentation. The resulting representations are classified using multiple ImageNet-pretrained architectures, where VGG19 yields the most consistent performance. **Results:** Experiments on the publicly available DDTI dataset show that BT-based preprocessing consistently improves performance over classical wavelet representations across multiple quadtree thresholds, with the best results obtained at T=30. Under this setting, the proposed *BT+TL* (VGG19) model achieves 98.91% accuracy, 98.11% sensitivity, 97.31% specificity, and a 98.89% F1-score, outperforming comparable approaches reported in the literature. **Conclusions:** Coupling geometry-adaptive transforms with modern TL backbones provides a robust and data-efficient strategy for ultrasound TN classification, particularly under limited annotation and challenging texture variability. The complete project is publicly available.

## 1. Introduction

Thyroid nodules (TNs) are commonly encountered, and their evaluation is predominantly performed using ultrasound (US), which provides a fast, non-invasive, and radiation-free imaging method. Due to its ability to generate real-time, high-resolution images of the thyroid gland, the US has become the primary diagnostic tool for assessing thyroid abnormalities [1,2]. However, traditional computer-aided diagnosis (CAD) systems used by radiologists for diagnosing TN relied on manually extracted features, such as shape, texture, and margin characteristics. The process of manual feature extraction is labour-intensive, inefficient, and operator-dependent, often introducing variability and limiting large-scale applicability in clinical settings [3]. Ongoing innovations in deep learning (DL) techniques have greatly enhanced CAD systems. However, when employing DL for TN diagnosis, several challenges arise. First, DL models typically require large labelled datasets for effective training, which are often scarce in the medical imaging domain due to privacy concerns, annotation costs, and limited expert availability. Additionally, training DL models on small datasets can lead to significant overfitting issues, potentially leading to poor generalisation on unseen data. Their black-box nature also presents a significant interpretability challenge, making clinical adoption difficult, as clinicians often require transparent and explainable decision-making processes. Lastly, variability in US images, originating from differing hardware settings and user-specific techniques, operator techniques, and patient-specific factors, further complicates model robustness and generalisation.

Transfer learning (TL) has emerged as a promising solution to address these limitations by leveraging pretrained models, initially trained on large-scale, general-purpose datasets, and fine-tuning them on smaller, domain-specific datasets such as thyroid US images. This approach reduces the dependency on extensive labelled data, mitigates overfitting, and enhances model generalisation across diverse imaging conditions. TL also offers the advantage of significantly reducing computational costs and training time, making it more practical for clinical deployment [4,5].

Wavelet transform (WT) is an effective technique for processing US images, allowing multi-resolution analysis. It enhances feature extraction, noise reduction, and image details, improving diagnostic accuracy in medical imaging applications [6,7,8]. However, this classical WT often struggle to capture fine spatial details in US images, especially in thyroid imaging, due to their fixed frequency and resolution properties. These transforms fail to adapt to the varying features of the image, such as the heterogeneous texture of thyroid tissues or the complex, low-contrast boundaries between different tissue types. As a result, they kill complex geometries, causing discontinuities and loss of information, as they primarily operate in horizontal, vertical, and diagonal directions. This leads to suboptimal feature extraction, particularly in regions with small nodules or irregular structures, which are critical for accurate diagnosis [9,10,11].

This study trains a DL model using TL on a publicly available thyroid US image dataset. Pretrained architectures such as ResNet, DenseNet, and EfficientNet have been used, originally learned from large-scale datasets (e.g., ImageNet), and provide transferable feature representations suitable for medical imaging tasks with limited data [12,13]. In this work, the fine-tuned TL-based models are employed to classify TN as benign or malignant. The use of pretrained networks facilitates faster convergence and improved generalisation, which is critical for reliable TN diagnosis and clinical decision support [14]. Additionally, in the part of preprocessing, the authors focus on the use of second-generation wavelets, specifically bandlet transform (BT), which are designed to generate decorrelated coefficients, eliminate redundancy, preserve essential information, and struggle WT limitations. Specifically, BT are based on geometric principles and are adept at capturing complex structures that are often not apparent when using WT. In addition, the BT approach is able to model real-world data more effectively, particularly when the data is non-uniformly sampled, lies on curves, or exists in higher-dimensional spaces like surfaces and manifolds. This work makes a unique contribution by integrating BT with TL. As far as we know, no prior research has explored this particular combination in the context of thyroid cancer (TC) classification.

In summary, this work is characterised by the following:

1. **Methodological contributions:** This study introduces a novel geometry-aware framework that integrates the BT with TL for TC classification in medical imaging, particularly for US images. The proposed approach exploits the capability of BT to adaptively model local geometric structures through quadtree-driven directional analysis, enabling the extraction of anisotropic and spatially coherent features that are not captured by conventional wavelet representations. By coupling these geometry-adaptive features with deep TL backbones, the framework enhances feature representation and robustness under limited labelled data conditions.
2. **Experimental contributions:** Extensive experiments demonstrate that integrating BT with TL significantly improves the accuracy and reliability of TC classification compared with classical wavelet-based and standalone TL approaches. Several ImageNet-pretrained architectures are systematically evaluated to assess their generalisation capability on thyroid US images. The results identify the best-performing architecture among the tested models, highlighting its superior ability to extract discriminative geometric and textural features while reducing dependence on large annotated datasets.

An overview of the paper’s organisation is provided as follows: Section 2 examines prior research in the field that have utilised WT and TL, particularly in the context of TC diagnosis and classification. Section 3 presents the background and preliminaries necessary to understand the proposed approach, including the theoretical foundations of WT and TL. Section 4 describes the proposed methodology in detail, outlining the key steps of the algorithm. Section 5 discusses the experimental results, performance evaluation, and comparative analysis. The paper concludes in Section 6, which reflects on the main contributions and explores prospective research paths.

## 2. Related Work

In recent years, significant efforts have been directed toward diagnosing benign and malignant TC. While many studies initially relied on the fine-needle aspiration biopsy (FNAB) method, the increasing use of US imaging is driven by its accessibility and cost-effectiveness. A range of artificial intelligence-based approaches have emerged to enhance diagnostic accuracy. The related literature is organised below according to the artificial intelligence methodology employed.

Several studies have employed DL techniques for TC detection using imaging and clinical data. Vahdati et al. [15] propose a DL-based method combining YOLOv5 for detection and XGBoost for classification using transverse and longitudinal US views. Similarly, Wang et al. [16] introduce a model that integrates multimodal magnetic resonance imaging (MRI) data with clinical features to predict lymph node metastasis in papillary thyroid cancer. Gummalla et al. [17] develop a hybrid framework using a sequential convolutional neural networks (CNNs) and K-means clustering for classifying thyroid images. Chandana et al. [18] present a deep CNN model for classifying adenoma, thyroiditis, and cancer based on computed tomography (CT) and US scans. Wang et al. [19] utilise a combination of binary logistic regression (BLR) and CNN for metastasis prediction, integrating genetic mutations and clinical data. Qi et al. [20] apply Mask R-CNN with ResNet-50 and feature pyramid network (FPN) for detecting gross extrathyroidal extension in TC, outperforming radiologists in accuracy. Finally, Zhang et al. [21] propose an automated DL-based system daptive WT-based AdaBoost algorithm (AWT-AA) to differentiate benign and malignant nodules in US imaging, supported by logistic regression analysis.

Other studies utilise ensemble learning (EL) to improve model robustness and generalisability. Shah et al. [22] design a deep ensemble model incorporating long short-term memory (LSTM), GRU, and Bi-LSTM for mutation detection in thyroid adenocarcinoma, achieving high diagnostic accuracy from genomic data. Zhang et al. [23] introduce MC-CNNs along with a weighted averaging ensemble and Faster Apriori for multi-view medical image classification and association rule mining. Zhang et al. [24] develop a dynamic ensemble TL-based system that integrates multi-view ultrasonography data, featuring a novel weighting mechanism for optimal decision-making.

TL has been applied in several works to enhance generalisation on medical datasets. Chen et al. [25] utilise an improved GoogLeNet model with secondary TL, total variation-based image restoration, and joint training on hospital and public datasets to classify TN. Ma et al. [26] introduce Mul-DenseNet, a multi-channel DenseNet model pretrained on ImageNet to simultaneously segment thyroid and breast lesions in US images. Bakht et al. [27] apply fine-tuned VGG-19 and AlexNet models with a weighted classification layer to cytology slides, enhancing performance despite class imbalance.

Hybrid and domain-specific architectures have also been explored. Lu et al. [28] propose a dual-tree complex WT-based CNN for segmenting human thyroid optical coherence tomography (OCT) images. The model uses wavelet pooling to preserve texture details and resist noise, improving segmentation robustness. Wang et al. [29] present a soft-label fully convolutional network (SL-FCN), enabling more accurate boundary delineation in TC segmentation tasks compared to hard-label models.

To the best of the authors’ knowledge, no existing study has investigated a geometry-aware BT combined with TL for medical image diagnosis or classification. As discussed above and summarised in Table 1, prior works employ DL, wavelet-based features, or TL independently, whereas the proposed framework uniquely integrates geometry-aware BT, TL, and CNNs to improve directional feature representation and TC discrimination.

## 3. Preliminaries

### 3.1. Wavelet Transform

WT is a powerful mathematical tool used in image processing to analyse images at multiple resolutions. Unlike the Fourier transform, which provides only frequency information, WT captures both spatial and frequency characteristics, making it highly effective for feature extraction. WT is based on the concept of analysing a signal or an image using scaled and shifted versions of a finite-duration function called the mother wavelet. This function is designed to be localised in both time and frequency domains, making wavelets suitable for capturing transient, localised, and multiscale features within signals and images. The fundamental principle of WT relies on the decomposition of a given function into a set of basis functions derived from a mother wavelet through scaling and translation operations. Mathematically, a wavelet function ψ(t) satisfies the admissibility condition, which ensures that it has zero mean and is well-localised:

(1) $$\int_{-\infty }^{+\infty }\psi \left(t\right)dt=0.$$

The wavelet basis functions are constructed by modifying the scale and position of the mother wavelet as follows:

(2) $$\psi_{a,b}\left(t\right)=\frac{1}{\sqrt{\left|a\right|}}\psi \left(\frac{t-b}{a}\right),$$

where *a* is the scaling parameter that controls the frequency resolution and *b* is the translation parameter that shifts the wavelet function in time. The continuous WT is given by

(3) $$W\left(a,b\right)=\int_{-\infty }^{+\infty }f\left(t\right)\frac{1}{\sqrt{\left|a\right|}}\psi \left(\frac{t-b}{a}\right)dt,$$

where W(a,b) represents the wavelet coefficients at different scales and positions. The discrete wavelet transform (DWT), which is a computationally efficient variant of continuous WT, employs dyadic scales ($a=2^{j}$) and integer translations ($b=k2^{j}$) to construct the wavelet basis. This results in an orthogonal or biorthogonal representation that allows hierarchical decomposition of images into different frequency subbands. Using the DWT, an image is decomposed into four distinct frequency subbands: LL, LH, HL, and HH. The LL subband retains the approximation information, primarily reflecting the image’s coarse details, while LH, HL, and HH contain detailed coefficients corresponding to horizontal, vertical, and diagonal details, respectively [9,31].

This process is mathematically represented by the system of equations in (4), where I(x,y) denotes the original image. The two-dimensional DWT is carried out by first applying the one-dimensional DWT along the rows, followed by its application along the columns.

(4) $$\begin{array}{cc} I_{\mathrm{LL}}\left(x,y\right) & =\sum_{m}\sum_{n}I\left(m,n\right)\phi \left(x-m\right)\phi \left(y-n\right)\\ I_{\mathrm{LH}}\left(x,y\right) & =\sum_{m}\sum_{n}I\left(m,n\right)\phi \left(x-m\right)\psi \left(y-n\right)\\ I_{\mathrm{HL}}\left(x,y\right) & =\sum_{m}\sum_{n}I\left(m,n\right)\psi \left(x-m\right)\phi \left(y-n\right)\\ I_{\mathrm{HH}}\left(x,y\right) & =\sum_{m}\sum_{n}I\left(m,n\right)\psi \left(x-m\right)\psi \left(y-n\right) \end{array}$$

where ϕ(x) is the scaling function, which generates the LL subband (approximation coefficients), capturing the coarse image details; ψ(x) is the wavelet function, which generates the LH, HL, and HH subbands (detailed coefficients). This decomposition provides a multi-resolution representation of the image, allowing for efficient image processing techniques such as feature extraction.

In addition, the application of the WT to thyroid US images faces several intrinsic challenges related to speckle noise, low contrast, and device dependence. Although wavelet decomposition separates images into multiple frequency bands, the strong and signal-dependent nature of speckle noise often overlaps with diagnostically relevant high-frequency components, making it difficult to suppress noise without simultaneously removing fine structural details of TNs [32]. This trade-off may lead to loss of edge information and texture cues that are critical for reliable segmentation and classification. Furthermore, the inherently low contrast between normal thyroid tissue and nodules limits the effectiveness of wavelet-based enhancement, as subtle intensity variations may not be sufficiently amplified across all decomposition scales. Device-dependent variability in US acquisition (e.g., probe frequency, gain settings, and imaging protocols) introduces inconsistent frequency distributions, which can degrade the robustness and generalisation of wavelet-based features across datasets. In addition, the performance of WT-based approaches is highly sensitive to the choice of mother wavelet, decomposition level, and thresholding strategy, requiring careful tuning that may not transfer well between different clinical environments. These challenges highlight the difficulty of relying solely on WT for thyroid US analysis and motivate the integration of more adaptive learning-based frameworks [33,34].

### 3.2. Bandlet Transform

The bandlet transform, introduced by Le Pennec and Mallat [35], is designed to construct a basis that aligns with the geometric structure of an image by locally deforming the spatial domain. This deformation simplifies the structure into a separable basis along a fixed direction—either horizontal or vertical. A key aspect of this transform is the *flow–curve relationship*, where the flow in the vertical direction corresponds to curves with non-vertical tangents. This allows for the construction of test bandelets that respect the geometric regularity of each sub-block. Mathematically, the basis function is defined as

(5) $$\tau \left(x\right)=\frac{1}{\sqrt{1+|c^{\prime }{\left(x\right)|}^{2}}}\left(\begin{array}{c} 1\\ c^{\prime }\left(x\right) \end{array}\right)$$

where c(x) defines the curve, and $c^{\prime }\left(x\right)$ denotes its slope, interpreted as the optical flow.

The bandlet transform is applied depending on the presence of geometric flow: if a sub-block exhibits no significant geometric variation, it is treated as uniformly regular and processed using a classical separable wavelet basis. Conversely, if geometric variations are detected, bandelet processing is employed. In cases involving singularities, additional Lagrangian-based computations are required.

The cost function governing this transformation is given by

(6) $$L\left(f,R,B\right)={∥f-f_{R}∥}^{2}+\lambda T^{2}\sum_{j}\left(R_{jG}+R_{jB}\right)$$

where *f* is the original image, $f_{R}$ is its reconstructed approximation, $R_{jG}$ denotes the number of bits used to encode the geometric flow (optical flow) in sub-block *j*, and $R_{jB}$ corresponds to the bits used to encode the quantised bandelet coefficients. The parameter λ is a Lagrangian multiplier that balances rate and distortion, and *T* is the quantisation step.

To efficiently represent the image, a quadtree decomposition is employed. This technique recursively divides the image domain into four quadrants (sub-blocks), denoted as $S_{1},S_{2},S_{3},$ and $S_{4}$, yielding a hierarchical representation. For each block *S*, the goal is to choose the best representation strategy—either by encoding *S* as a whole or by subdividing it further. This decision is governed by minimising the Lagrangian cost:

(7) $$L_{0}\left(S\right)=\min\left\{L_{\mathrm{direct}}\left(S\right),\tilde{L}\left(S\right)\right\}$$

Here, $L_{\mathrm{direct}}\left(S\right)$ represents the cost of encoding the block *S* directly without further subdivision, while $\tilde{L}\left(S\right)$ is the cumulative cost of encoding its four children:

(8) $$\tilde{L}\left(S\right)=L_{0}\left(S_{1}\right)+L_{0}\left(S_{2}\right)+L_{0}\left(S_{3}\right)+L_{0}\left(S_{4}\right)+\lambda T^{2}$$

The term $\lambda T^{2}$ accounts for the overhead cost of subdivision. This recursive strategy ensures that each sub-block is processed in the most efficient manner, adapting the representation complexity to local image content. To address curved singularities in image structures, a deformation operator is applied to locally realign blocks so that anisotropic features are better captured along either horizontal or vertical directions. This leads to a new orthonormal basis in $L^{2}\left(\Omega \right)$, replacing the standard horizontal wavelets $H_{n,j}\psi$ with geometry-adapted functions defined as

(9) $$\left(\begin{array}{c} \phi_{j,j_{1}}\left(x_{1}\right)\psi_{j,j_{2}}\left(x_{2}-c\left(x_{1}\right)\right),\psi_{j,j_{1}}\left(x_{1}\right)\phi_{j,j_{2}}\left(x_{2}-c\left(x_{1}\right)\right),\psi_{j,j_{1}}\left(x_{1}\right)\psi_{j,j_{2}}\left(x_{2}-c\left(x_{1}\right)\right) \end{array}\right)$$

Here, $x_{1}$ and $x_{2}$ represent the horizontal and vertical spatial coordinates of the image domain, respectively. The function $c(x_{1})$ defines a local geometric flow or deformation that aligns the vertical coordinate $x_{2}$ with directional image features such as edges. By warping the coordinate system through $x_{2}-c\left(x_{1}\right)$, the basis functions adapt to the underlying structure of the image, improving alignment with anisotropic features. This transformation process, known as *bandeletisation*, builds an orthonormal basis that is aligned with the image’s geometric structure. By doing so, it replaces traditional wavelet bases with more expressive functions, allowing for enhanced compression and analysis by better respecting geometric regularities in both horizontal and vertical orientations.

(10) $$\left(\begin{array}{c} \psi_{j,j_{1}}\left(x_{1}\right)\psi_{j,j_{2}}\left(x_{2}-c\left(x_{1}\right)\right)\\ \psi_{j,j_{1}}\left(x_{1}\right)\phi_{j,j_{2}}\left(x_{2}-c\left(x_{1}\right)\right)\\ \psi_{j,j_{1}}\left(x_{1}\right)\psi_{j,j_{2}}\left(x_{2}-c\left(x_{1}\right)\right) \end{array}\right)=\left(\begin{array}{c} \psi_{j,j_{1}}^{H}\\ \psi_{j,j_{1}}^{V}\\ \psi_{j,j_{1}}^{D} \end{array}\right)\left(j,l\right)\;n_{1},n_{2},$$

The theoretical foundation of relies on their ability to optimise representation in anisotropic geometric image structures, unlike traditional WT that only capture local oscillations. This geometric adaptability enables improved feature extraction, making bandelets particularly effective for DL applications where efficient hierarchical feature representation is crucial [18]. The final output of the bandlet transform consists of bandelet coefficients that capture the image’s directional and structural information. These coefficients, derived from optimal sub-block partitioning and adaptive transformations, form the bandelet feature vector, which is fed into a DL model. The bandelet feature vector includes multiscale directional energy distributions, geometric flow descriptors, and localised structural patterns, providing a compact yet expressive representation of the image. These bandelet features are then input into CNNs, or transformer-based models, depending on the application. The hierarchical nature of bandelet-based feature extraction ensures that DL models receive a structurally enriched representation of the data, significantly improving their performance in image classification, segmentation, super-resolution, and medical image analysis. By leveraging the BT’s ability to adapt to image geometry, DL architectures can process images more efficiently, achieving higher accuracy while reducing computational overhead [6,36].

### 3.3. Inductive TL

Inductive TL is a machine learning paradigm where a model trained on a source task is adapted to a target task, assuming that both tasks share some structural similarities while differing in their label spaces or distributions. Mathematically, given a labelled source dataset $D_{S}={\left\{\left(x_{i}^{S},y_{i}^{S}\right)\right\}}_{i=1}^{N_{S}}$ associated with a task $T_{S}$ and a labelled target dataset $D_{T}={\left\{\left(x_{j}^{T},y_{j}^{T}\right)\right\}}_{j=1}^{N_{T}}$ associated with a task $T_{T}$, the goal is to learn a target function $f_{T}:X_{T}\to Y_{T}$ by leveraging knowledge from a source function $f_{S}:X_{S}\to Y_{S}$, where $X_{S}=X_{T}$ but $P\left(X_{S},Y_{S}\right)\neq P\left(X_{T},Y_{T}\right)$, meaning that while both tasks share a common feature space, they exhibit differences in their distributions or label mappings. The learning process consists of two key steps: (i) pretraining and (ii) fine-tuning. During pretraining, a model $f_{S}\left(x;\theta_{S}\right)$ is trained on the source dataset to minimise the loss function:

(11) $$\theta_{S}^{*}=\arg\min_{\theta }\sum_{i=1}^{N_{S}}L\left(y_{i}^{S},f_{S}\left(x_{i}^{S};\theta \right)\right)$$

where L refers to the loss function appropriate to the task at hand, typically cross-entropy for classification or mean squared error for regression. The learned parameters $\theta_{S}^{*}$ serve as the initialisation for the target model $f_{T}\left(x;\theta_{T}\right)$, which is then fine-tuned on the target dataset using

(12) $$\theta_{T}^{*}=\arg\min_{\theta }\sum_{j=1}^{N_{T}}L\left(y_{j}^{T},f_{T}\left(x_{j}^{T};\theta \right)\right)$$

Typically employing strategies such as feature extraction, where early layers of the pretrained model are frozen while only task-specific layers are updated, or full fine-tuning, where all model parameters are updated but with a lower learning rate to preserve generalizable knowledge. Optimisation is commonly performed using gradient descent with an update rule $\theta_{T}\leftarrow \theta_{T}-\alpha \nabla_{\theta_{T}}L_{T}$, where α is the learning rate, ensuring stable adaptation to the new task. To balance knowledge retention and task-specific adaptation, a weighted loss function $L=\lambda L_{S}+\left(1-\lambda \right)L_{T}$ can be employed, where λ controls the contribution of the source knowledge during training. Inductive TL is widely applied in DL, particularly in computer vision, where CNNs such as ResNet, VGG, or EfficientNet pretrained on ImageNet are fine-tuned for specialised applications like medical image analysis, object detection, or satellite imagery classification, as well as in natural language processing [8].

## 4. Methodology

This section evaluates the performance of both WT and approaches in enhancing image classification and feature extraction accuracy within the proposed framework. The algorithm is specifically designed to differentiate between malignant and benign TN, aiming to improve diagnostic precision by leveraging geometry-adaptive representations.

**Figure 1:**
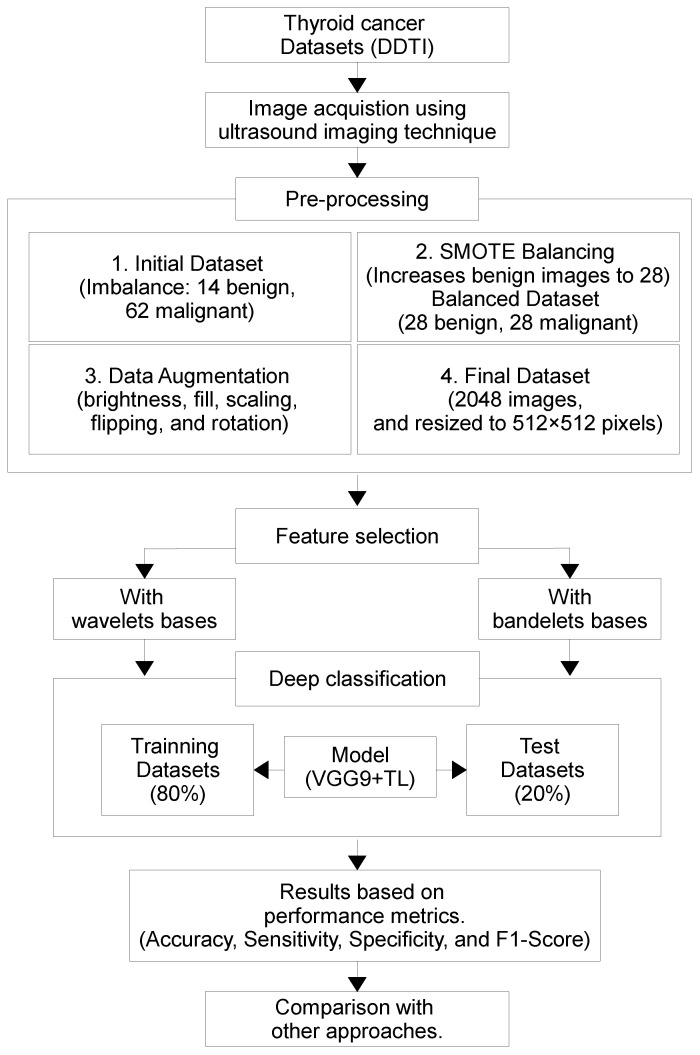
Schematic representation of the study’s methodology.

The overall procedural workflow is illustrated in Figure 1. The process begins with the digital database of thyroid ultrasound images (DDTI) dataset, where US images are acquired and preprocessed. Due to class imbalance, synthetic minority oversampling technique (SMOTE) is applied to augment benign cases and create a balanced dataset, followed by additional data augmentation (DA) techniques to further enhance data diversity. Feature selection is then performed using both WT and bases, allowing extraction of rich structural and texture descriptors. These features are subsequently fed into a DL classifier using TL from the pretrained VGG19 model. The dataset is split into training and testing subsets, and the classification model is trained accordingly. Performance is evaluated using key metrics and compared against alternative methods to assess the effectiveness and efficiency of the proposed approach. Algorithm 1 outlines the suggested TN classification algorithm based on DL techniques.
**Algorithm 1** TN Classification Algorithm 1: **function** LoadDatasets 2:       Dataset ← DDTI▹ 134 images: 14 benign, 62 malignant 3:       **return** Dataset 4: **end function** 5: **function** Preprocess(Dataset) 6:       Benign ← GetBenignImages(Dataset)▹ 14 images 7:       Malignant ← GetMalignantImages(Dataset)▹ 62 images 8:       New_Benign ← SMOTE (Benign, target = 28)▹ Oversample benign 9:       Balanced_Dataset ← Combine(New_Benign, Malignant[0:28])10:      Augmented_Dataset ← Augment(Balanced_Dataset, Techniques = {Brightness, Flip, Rotate, Resise_to_512 × 512})11:      **return** Augmented_Dataset▹ Target: 2048 images12: **end function**13: **function** ExtractFeatures(Augmented_Dataset)14:      Features ← []15:      **for** each image **in** Augmented_Dataset **do**16:            Bandelet_Features ← ApplyBandeletTransform(image)17:            Features ← Add(Bandelet_Features)18:      **end for**19:      **return** Features20: **end function**21: **function** Classify(Augmented_Dataset, Features)22:      Train_Data ← Take80Percent(Augmented_Dataset)▹ 1638 images23:      Val_Data ← Take20Percent(Augmented_Dataset)▹ 410 images24:      Models ← {VGG16}▹ Simplified list25:      **for** each model **in** Models **do**26:            LoadPretrained(model)27:            FineTune(model, Train_Data, Features)28:            Predictions ← Test(model, Val_Data)29:            Save(Predictions)30:      **end for**31:      **return** Predictions32: **end function**33: **function** Evaluate(Predictions, Val_Data)34:      **for** each model **in** Predictions **do**35:            Accuracy ← CalculateAccuracy(Predictions, Val_Data)36:            Sensitivity ← CalculateSensitivity(Predictions, Val_Data)37:            Display(model, Accuracy, Sensitivity)38:      **end for**39: **end function**40: **function** Main41:      Dataset ← LoadDatasets()42:      Augmented_Dataset ← Preprocess(Dataset)43:      Features ← ExtractFeatures(Augmented_Dataset)44:      Predictions ← Classify(Augmented_Dataset, Features)45:      Evaluate(Predictions, Augmented_Dataset)46: **end function**

### 4.1. Input TC Datasets

**Figure 2:**
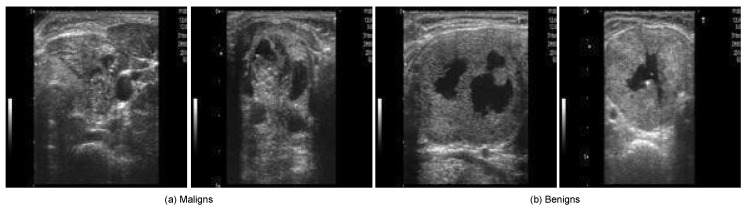
TC images samples from the DDTI dataset.

The DDTI dataset [37], provided by the Universidad Nacional de Colombia and the Instituto de Diagnóstico Médico (IDIME), is an open-access collection of thyroid US images. Table 2 summarises its key characteristics. This dataset was selected as the primary source for this study due to its credibility, public availability, and relevance to TN classification tasks. Previously used in related research, such as in [38], it serves as a reliable benchmark for performance comparison. Its open-access nature addresses the common challenge of restricted medical datasets, which often require complex ethical approvals. Additionally, the dataset is well-annotated, with clear labels distinguishing between benign and malignant cases, ensuring suitability for supervised learning. Although limited in size, the DDTI dataset remains valuable given the scarcity of publicly available, high-quality thyroid US datasets. Figure 2 illustrates sample thyroid US images from the DDTI dataset, showcasing both malignant and benign cases.

### 4.2. Preprocessing

To tackle dataset imbalance in TC classification, a two-step approach was applied: synthetic oversampling followed by DA [39,40]. Initially, the dataset comprised 14 benign and 62 malignant images, resulting in a significant class imbalance that could bias model predictions. To mitigate this, the SMOTE was employed to generate additional synthetic benign images by interpolating between existing samples, increasing their count from 14 to 28. This adjustment ensured parity with the 28 malignant images, creating a more balanced dataset for training. Following dataset balancing, various DA techniques were implemented to further expand the dataset to 2048 images, introducing variability to enhance model generalisation and robustness. The augmentation process included brightness adjustments to simulate diverse lighting conditions, ensuring the model’s adaptability to varying illumination levels. Nearest-neighbour fill was applied to preserve pixel integrity during transformations, preventing unwanted artefacts. Height scaling was introduced to modify the vertical proportions of images, while horizontal flipping effectively doubled spatial variations. Rotation was employed to alter image orientation, preventing model bias toward specific angles. This preprocessing not only mitigated class imbalance but also enriched dataset diversity, reducing overfitting risks and enhancing the model’s ability to accurately classify benign and malignant cases. By integrating SMOTE with targeted DA, the dataset became more representative and robust, ultimately leading to improved generalisation and performance in TC classification.

Prior to applying the BT, all US images are resized to a fixed spatial resolution of 512×512 pixels and subsequently downsampled to 224×224 pixels to match the input size of the VGG19 architecture. After the Bandelet decomposition, normalisation is applied independently to each coefficient channel using z-score normalisation:

(13) $$\hat{x}=\frac{x-\mu }{\sigma },$$

where μ and σ denote the mean and standard deviation of the bandelet coefficients computed over the training set. This normalisation step ensures comparable dynamic ranges across all channels and stabilises network training.

### 4.3. Bandelet Feature Selection

**Figure 3:**
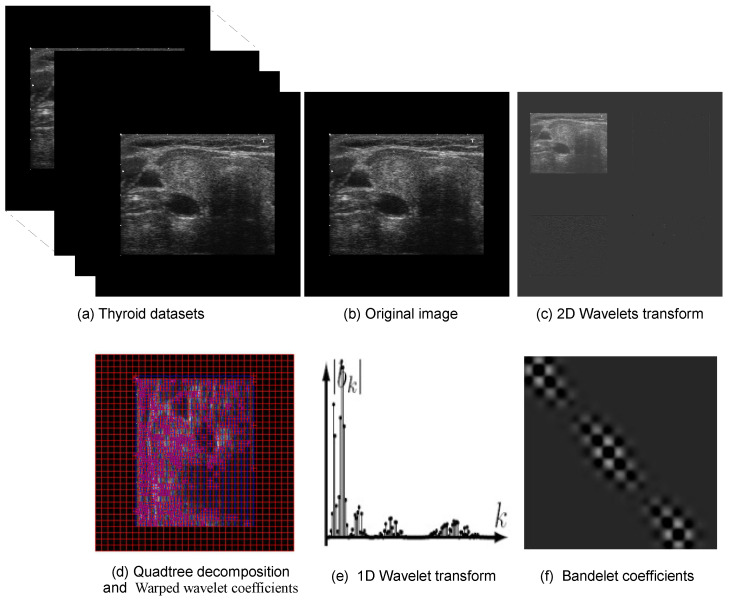
The steps employed in the proposed DL-based TC scheme.

The proposed feature extraction strategy exploits the intrinsic geometric structure of thyroid US images in order to enhance the encoding of directional and textural information for classification. Empirical studies demonstrate that the classification performance of TC images improves significantly when using TL-extracted features compared to WT [41]. Figure 3 illustrates the main processing stages of the proposed framework for thyroid US image analysis. Starting from TN images acquired from the DDTI dataset, the images are transformed into a geometry-aware representation that emphasises structural and directional characteristics. The resulting bandelet coefficients provide a compact and discriminative description of complex and anisotropic patterns in TNs, which is subsequently exploited by the DL classifier for robust feature learning.

After applying the BT to each preprocessed US image, the resulting representation consists of geometry-adapted coefficient maps obtained by deforming wavelet bases along locally estimated geometric flows using quadtree-based partitioning. Four dominant bandelet coefficient channels are retained, including one approximation component and three directionally adapted detail components that encode horizontal, vertical, and diagonal geometric structures. Unlike conventional wavelet subbands, these bandelet coefficients preserve anisotropic edges and curved contours more effectively, capturing the most discriminative geometric and textural information relevant to TN characterisation, such as boundaries, contours, and spatially coherent patterns. These four bandelet coefficient maps are stacked along the channel dimension to form a multi-channel tensor compatible with CNN input requirements.

Finally, the normalised four-channel bandelet tensor is provided as input to the CNN in place of the original greyscale US image. This enables the network to learn directly from geometry-aware frequency representations rather than raw pixel intensities, allowing the deep model to exploit directional, structural, and textural cues encoded by the BT for improved TN classification.

### 4.4. Deep Classification

Medical image classification benefits considerably from the adoption of the proposed strategy, where acquiring large, well-annotated datasets is time-consuming and resource-intensive [8,42]. Typically, a network trained on a large dataset like ImageNet is either fine-tuned by adjusting its layers or used as a fixed feature extractor, transferring learned representations to the new task. To optimise training and evaluation, the dataset was carefully partitioned following DL best practices, with 80% (1638 samples) allocated for training and 20% (410 samples) designated for validation. The training subset was essential for refining model parameters, improving the ability to extract meaningful feature representations, while the validation subset played a crucial role in ensuring generalisation and detecting overfitting by evaluating the model’s performance on unseen data.

**Figure 4:**
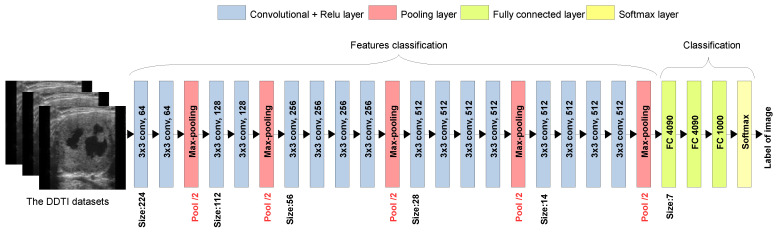
The typical set of layers in a pretrained VGG19 model.

The VGG19 architecture, illustrated in Figure 4, is employed in this study as a pretrained model for TL. It consists of a structured DL model used for feature extraction and classification. It begins with an input image (from the DDTI dataset) and processes it through multiple convolutional layers with 3 × 3 filters and ReLU activation, which capture hierarchical features such as edges, textures, and patterns. After every two or four convolutional layers, a max pooling layer (red) is applied to reduce spatial dimensions while preserving essential features. The model progressively increases the number of filters from 64 to 512, allowing deeper layers to extract more complex representations. Once feature extraction is complete, the fully connected layers flatten the extracted features and pass them through two dense layers with 4096 neurons, followed by a Softmax layer that classifies the image into 1000 possible categories. In TL, the convolutional base is typically frozen, and the fully connected layers are modified or fine-tuned for new classification tasks, making VGG19 highly effective for our classification of TC images [43]. By leveraging TL, these models could retain valuable pre-learned representations, minimising the data and computational resources required for training, a crucial advantage in medical imaging applications where data scarcity is a challenge. In TL, modifying layers between a pretrained model and a proposed model involves carefully adapting the network architecture to balance feature reuse and task-specific learning. First, the pretrained model, typically a deep neural network VGG, is loaded, and its architecture is examined. The earlier convolutional layers embedding layers in transformers, which capture fundamental and transferable features, are usually frozen to retain their learned representations. The fully connected (dense) layers or task-specific heads in natural language processing models, which encode high-level, domain-specific features, are removed or replaced with new layers customised for the target task. This replacement often involves adding new dense layers, batch normalisation, and dropout (to prevent overfitting), incorporating an output layer that uses task-specific activation functions, like softmax for multi-class classification and sigmoid for binary cases. Fine-tuning can be applied to some middle layers if the new dataset is sufficiently large, gradually unfreezing layers while using a lower learning rate to prevent catastrophic forgetting of previously learned features. Additionally, TL methods such as feature extraction (where only new layers are trained) or full fine-tuning (where pretrained weights are adjusted) are selected based on dataset sise, computational power, and the similarity between the source and target domains. The modified model is then compiled and trained, leveraging techniques like learning rate scheduling and data augmentation to improve adaptation while ensuring that the knowledge from the pretrained model enhances the performance of the new task.

### 4.5. Performance Metrics

Common metrics are used for evaluating US-based TC classification. These metrics offer a thorough assessment of the model’s performance in differentiating between benign and malignant TNs. The *Accuracy* (*Acc*) measures the overall correctness of predictions. As a harmonic mean of precision and recall, the F1 Score serves as a comprehensive metric for evaluating classification performance, especially in imbalanced datasets:

(14) $$\mathrm{Acc}\left(\%\right)=\frac{\mathrm{TP}+\mathrm{TN}}{\mathrm{TP}+\mathrm{FP}+\mathrm{TN}+\mathrm{FN}}\times 100,\;\;\;\;\;\;\;\;\;\;\;\;\;\;\;\;F1\left(\%\right)=2\times \frac{\mathrm{Precision}\times \mathrm{Recall}}{\mathrm{Precision}+\mathrm{Recall}}\times 100$$

*Sensitivity* (*Sen*) quantifies the proportion of actual malignant cases correctly identified, *Specificity* (*Spe*) measures the proportion of benign cases correctly classified, and *Precision* (*P*) indicates the ratio of predicted malignancies that are confirmed as actual malignant instances:

(15) $$\mathrm{Sen}\left(\%\right)=\frac{\mathrm{TP}}{\mathrm{TP}+\mathrm{FN}}\times 100,\;\mathrm{Spe}\left(\%\right)=\frac{\mathrm{TN}}{\mathrm{TN}+\mathrm{FP}}\times 100,\;P\left(\%\right)=\frac{\mathrm{TP}}{\mathrm{TP}+\mathrm{FP}}\times 100$$

These metrics collectively ensure a reliable and multidimensional assessment of classification performance. The abbreviations TP, TN, FP, and FN refer to true positives, true negatives, false positives, and false negatives in that order.

## 5. Results and Discussion

### 5.1. Experiments

**Figure 5:**
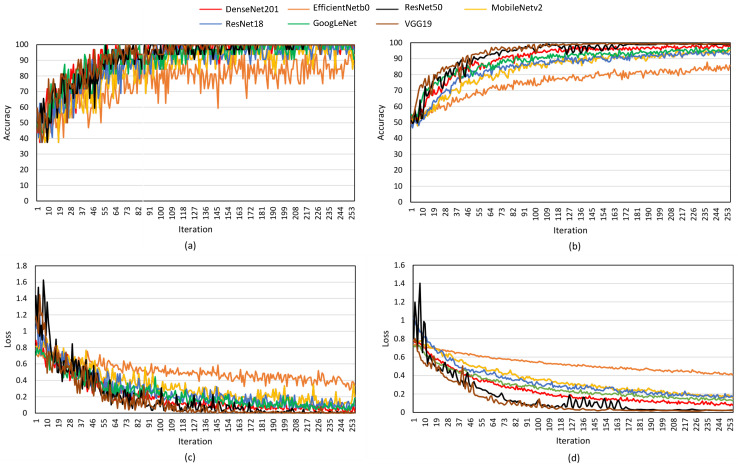
DL classification performance based on BT+TL (VGG19) for TC images classification. (**a**) Training accuracy; (**b**) validation accuracy; (**c**) training loss; (**d**) validation loss.

The obtained training and validation accuracy, along with their corresponding losses for different DL models, are illustrated in Figure 5. To justify the adoption of VGG19 in this study, multiple pretrained models were assessed and compared. The obtained results demonstrate that VGG19 consistently outperforms other models in TC classification using the BT+TL approach. The training accuracy curve (Figure 5a) shows that VGG19, ResNet50, and DenseNet201 achieve rapid convergence, surpassing 90% accuracy early in the training process, while MobileNetV2, EfficientNetB0, and GoogLeNet exhibit slower improvements and lower final accuracy values. Similarly, the validation accuracy graph (Figure 5b) confirms that VGG19 maintains the highest accuracy, followed by ResNet50 and DenseNet201, indicating strong generalisation to unseen data. In contrast, MobileNetV2 and GoogLeNet display lower validation accuracy, suggesting difficulties in capturing complex patterns in TC images. The training loss curve (Figure 5c) illustrates a steady decrease across all models, with VGG19 and ResNet50 achieving the lowest final loss values, reflecting their ability to minimise classification errors efficiently. However, GoogLeNet and MobileNetV2 maintain relatively higher loss values, implying weaker learning performance. The validation loss curve (Figure 5d) further supports these findings, as VGG19, ResNet50, and DenseNet201 exhibit smooth, consistently decreasing validation loss, whereas MobileNetV2 and GoogLeNet show more fluctuations, suggesting overfitting or instability during validation. These results highlight the superior performance of VGG19, which not only achieves the highest accuracy but also demonstrates better stability, faster convergence, and lower loss values, making it the most effective model for TC classification in this study.

In Table 3, the performance of the WT and BT is compared under varying quadtree decomposition thresholds (*T*) across four evaluation metrics: accuracy, sensitivity, specificity, and F1 Score. The WT serves as a strong baseline, achieving an accuracy of 0.9430, sensitivity of 0.9302, specificity of 0.9433, and an F1 Score of 0.9584. When the bandlet is applied, performance improves at lower thresholds (T=10), with enhanced accuracy (0.9511), sensitivity (0.9622), specificity (0.9774), and F1 Score (0.9807) compared to the WT. As the threshold increases to T=20, both accuracy (0.9635) and F1 Score (0.9645) continue to rise; however, sensitivity decreases to 0.9108, indicating a reduced ability to identify true positives. The optimal performance for the is observed at T=30, yielding the highest accuracy (0.9891), sensitivity (0.9811), and F1 Score (0.9889), while maintaining a strong specificity of 0.9731.

For the BT, the threshold value T=30 used in the quadtree decomposition was determined empirically based on the Lagrangian cost function defined in the theoretical framework. Since the threshold controls the stopping criterion for region subdivision according to homogeneity and segmentation cost, several candidate values of *T* were evaluated by minimising the Lagrangian cost while monitoring segmentation accuracy and computational complexity. The value T=30 achieved the best trade-off between accurate region partitioning and limited over-segmentation, while avoiding excessive computational burden. This selection links the theoretical cost formulation with the experimental setup and ensures both stable convergence and good generalisation performance.

In Table 4, the performance of the proposed BT+TL (VGG19) model is compared with several recent state-of-the-art approaches evaluated on the same dataset. Gummalla et al. [17] achieved an accuracy of 0.8150 and sensitivity of 0.8310, with a high precision of 0.9740. Their method focused on enhancing TN classification using DL-based feature extraction; however, the relatively low accuracy and incomplete reporting of specificity indicate limitations in achieving balanced classification performance, particularly in reducing false negatives and false positives simultaneously. Zhang et al. [23] reported a high accuracy of 0.9870 using a DL framework for thyroid image analysis. Although this result demonstrates strong classification capability, the absence of sensitivity, specificity, and precision metrics makes it difficult to comprehensively assess the robustness and clinical reliability of their model, especially in terms of error distribution across malignant and benign classes. Sharma et al. [30] obtained an accuracy of 0.9283, specificity of 0.8889, and precision of 0.8776 by integrating an IoT-assisted DL system for medical image classification. While their approach shows reasonable performance, the lower specificity and precision compared to the proposed model suggest limited discrimination capability, particularly in challenging or borderline thyroid cases. In contrast, the proposed BT+TL (VGG19) framework achieves the highest overall performance, with an accuracy of 0.9891, sensitivity of 0.9811, specificity of 0.9731, and precision of 0.9968. These results demonstrate a consistent improvement over existing methods across all reported metrics. The integration of BT enables the model to effectively capture complex geometric and spatial patterns in thyroid US images, while TL with VGG19 enhances feature generalisation and reduces dependency on large labelled datasets. This synergistic design yields superior discriminative capability and robustness, making the proposed model more suitable for reliable clinical thyroid classification.

### 5.2. Statistical Performance Analysis

To ensure the statistical reliability of the reported results, all experiments were conducted using repeated *k*-fold cross-validation. For each performance metric, descriptive statistics including the mean, median, variance, standard deviation, quartiles, as well as the minimum and maximum values, were computed across folds. The proposed BT and TL framework based on VGG19 achieved a high mean training accuracy of 98.90% and a closely matched validation accuracy of 98.85%, with low standard deviations (1.75 and 0.69, respectively), demonstrating strong stability and consistency across different data partitions. The observed training accuracy ranged from 87.5% to 100%, while validation accuracy varied within a narrower interval from 93.66% to 99.51%, indicating robust performance even under the least favourable data splits.

Likewise, both training and validation losses exhibit low mean values (0.0325 and 0.0351) with limited dispersion, as reflected by their small variances and narrow interquartile ranges. The minimum training and validation losses reached 0.0006 and 0.0133, respectively, whereas their maximum values remained bounded at 0.3298 and 0.1472, further confirming stable convergence behaviour and the absence of extreme outliers. Overall, the limited spread between minimum and maximum values, together with the tight quartile distributions, confirms the generalisation capability of the proposed model without evidence of overfitting.

### 5.3. Clinical Implications and Decision Support

From a clinical perspective, the proposed BT+TL framework is designed to function as a CAD support tool for thyroid US examination rather than as a replacement for radiologists. In a realistic clinical workflow, the US image acquired during routine examination can be automatically processed by the proposed system to provide a probability score indicating benign or malignant thyroid nodules, together with visual feature representations derived from geometry-aware Bandelet coefficients.

Such a system can assist radiologists in several ways. First, it can serve as a second-opinion tool to reduce diagnostic uncertainty, particularly in borderline or visually ambiguous cases. Second, it can improve diagnostic consistency by reducing inter-observer variability among clinicians with different levels of experience. Third, it can help prioritise high-risk cases for further examination, biopsy, or specialist referral, thereby improving workflow efficiency.

The lightweight nature of the proposed pipeline and its reliance on standard US images make it suitable for integration into existing hospital information systems or US workstations without modifying current acquisition protocols. By supporting clinical decision-making with objective and reproducible predictions, the proposed model has the potential to enhance diagnostic accuracy while maintaining the radiologist as the final decision authority.

## 6. Conclusions

In this paper, we introduced a novel TN classification framework that tightly integrates geometric feature encoding via the BT with TL on a pre-trained VGG19 backbone. The core technical innovation lies in employing a quadtree-driven BT to locally adapt basis functions to the intrinsic flow of nodule contours, yielding a sparse set of directional coefficients that encode anisotropic texture and edge continuity more faithfully than standard wavelets. By systematically varying the quadtree subdivision threshold, we demonstrated that finer-scale geometric adaptivity optimises the trade-off between coefficient sparsity and reconstruction fidelity, directly translating into a marked improvement in classification metrics (98.91% accuracy, 98.11% sensitivity, 97.31% specificity, 98.89% F1 Score).

From a methodological standpoint, our study confirms three key findings: (i) Geometric versus separable bases: Replacing classical WT with BT yields a consistent gain across all metrics, underscoring the value of geometry-aware multiscale analysis in US images. (ii) Quadtree threshold optimisation: Intermediate thresholds strike the best balance, whereas overly coarse (T < 10) or overly fine (T > 50) partitions either underfit large-scale structures or overfit noise, respectively. (iii) TL strategy: Leveraging VGG19’s mid-level feature maps, frozen through the initial training epochs and then selectively unfrozen, accelerates convergence and mitigates overfitting on the limited DDTI dataset.

Looking forward, several avenues warrant exploration. First, extending the BT stage to incorporate adaptive Lagrangian rate-distortion optimisation could further refine coefficient selection under constrained bit budgets, facilitating on-device inference for portable US systems. Second, integrating vision Transformers as the downstream classifier may capitalise on the global attention mechanism to exploit further the long-range dependencies encoded by bandelet features. Finally, exploring semi-supervised or self-supervised pre-training on large unlabelled US repositories—augmented by synthetic data generated via generative adversarial networks—could improve generalisability and alleviate the label scarcity that continues to challenge medical imaging applications.

## Figures and Tables

**Table 1:** Summary and comparison of state-of-the-art TC approaches.

Ref.	Used Method	Datasets	BPM (%)	Limitations	Employing		
					WT	BT	TL
[15]	Multi-view DL	Private data	Sensitivity: 84.00 Specificity: 63.00 F1 Score: 76.00	Studies should validate the results on other imaging modalities	✗	✗	✗
[16]	AMMCNet	Private data	Accuracy: 85.70Sensitivity: 90.00 Specificity: 90.90	Small sample size and single-centre data	✗	✗	✗
[22]	Deep EL	Ensembl IntOGen	Accuracy: 96.00 Sensitivity: 92.00 Specificity: 100	Limited dataset may affect generalisability	✗	✗	✗
[17]	CNN with K-means	DDTI	Accuracy: 81.50 Precision: 97.40, Sensitivity: 83.10	Model performance depends on annotated data	✗	✗	✗
[18]	Deep CNN	Private data	Accuracy: 97.20	High computational process	✗	✗	✗
[23]	MC-CNNs	DDTI, UCI thyroid and private datasets	Acurracy: 98.70	The DL models require high computational power	✗	✗	✗
[29]	SL-FCN	DISH and FISH Breast, and Thyroid Dataset	Dice Score: 89.00	SL-FCN requires significant computational power	✗	✗	✗
[19]	BLR and CNN	Clinicopathological	AUC: 89.00	Using BLR but limited to retrospective data and needs validation on larger datasets	✗	✗	✗
[30]	DL and EL	DDTI	Accuracy: 92.83 Precision: 87.76 Specificity: 88.89	Relies on a single public dataset	✗	✗	✗
[20]	Mask R-CNN, ResNet-50 and FPN	Private data	Accuracy: 87.00 Sensitivity: 80.00 Specificity: 92.00	Single-province dataset and requiring broader validation	✗	✗	✗
[25]	GoogLeNet with TL	Hospital and public thyroid US images	Accuracy: 96.04 F1 Score: 98.74 Precision: 98.42	The model requires high computational power	✗	✗	✓
[26]	Multi-channel DenseNet	Private data	Accuracy: 92.57 Sensitivity: 98.69 F1 Score: 95.96	Dependence on high-quality annotations	✗	✗	✓
[24]	Dynamic ensemble TL	Private data	Accuracy: 93.00Specificity: 95.00 F1 Score: 93.00	Limited data sources	✗	✗	✓
[27]	Fine-tuned VGG-19	Private data	Accuracy: 93.05 Sensitivity: 92.90 F1 Score: 92.80	Limited comparison with other models	✗	✗	✓
[28]	WT-based CNN	Custom human thyroid OCT	Accuracy: 98.60	The model adds extra computations compared to traditional CNNs	✓	✗	✗
[21]	AWT-AA	Private data	Accuracy: 95.00, Sensitivity: 97.50 Specificity: 86.00	The study is based on US images from a single institution	✓	✗	✗

Abbreviations: Best performance metrics (BPMs); Wavelet transform (WT); Bandelet transform (BT); Transfer learning (TL).

**Table 2:** Characteristics of the DDTI Thyroid US Dataset.

Attribute	Description
Image Count	134 images
Image Format	PNG (some JPEG)
Image Resolution	560×315 pixels
Benign Cases	14 images
Malignant Cases	62 images
Image Modality	B-mode 2D greyscale US
Frame Rate	15–30 fps (derived from video sequences)
US Equipment	Toshiba Nemio 30 and Nemio MX
Transducer Types	12 MHz linear and convex transducers
Axial Resolution	0.1–0.15 mm
Lateral Resolution	0.5–1 mm
Penetration Depth	4–6 cm
Field of View	38–50 mm (linear), 60–80 mm (convex)
Dynamic Range	50–70 dB

**Table 3:** A comparison of results between BT and WT for different values of quadtree decomposition threshold (T).

Transform	Accuracy	Sensitivity	Specificity	F1
Wavelet	0.9430	0.9302	0.9433	0.9584
Bandelet (T = 10)	0.9511	0.9622	0.9774	0.9807
Bandelet (T = 20)	0.9635	0.9108	0.9802	0.9645
Bandelet (T = 30)	0.9891	0.9811	0.9731	0.9889
Bandelet (T = 40)	0.9803	0.9723	0.9897	0.9746
Bandelet (T = 50)	0.9787	0.9794	0.9825	0.9764

**Table 4:** A comparison of the performance between our proposed model and previous approaches using the same dataset.

Methods	Accuracy	Sensitivity	Specificity	Precision
[17]	0.8150	0.8310	-	0.9740
[23]	0.9870	-	-	-
[30]	0.9283	-	0.8889	0.8776
**Our BT+TL (VGG19)**	**0.9891**	**0.9811**	**0.9731**	**0.9968**

## Data Availability

Publicly available datasets were analysed in this study. The DDTI thyroid ultrasound dataset used in this work is publicly available (see Ref. [31]).

## Abbreviations

BT	bandelet transform
CNN	convolutional neural networks
US	ultrasound
DA	data augmentation
TC	thyroid cancer
TN	thyroid nodules
TL	transfer learning
WT	wavelet transform
DWT	discrete wavelet transform
BT	bandlet transform
DDTI	digital database of thyroid ultrasound images
BLR	binary logistic regression
FPN	feature pyramid network
SL-FCN	soft-label fully convolutional network
OCT	optical coherence tomography
SMOTE	synthetic minority oversampling technique
LSTM	long short-term memory
DL	deep learning
